# Pseudoaneurysm formation after embolization of giant arteriovenous malformation of the lower limb associated with Parkes Weber syndrome: A case report and literature review

**DOI:** 10.1016/j.radcr.2024.07.117

**Published:** 2024-08-10

**Authors:** Hiroto Yasumura, Koji Tao, Ryo Imada, Yushi Yamashita, Naoki Tateishi, Kenichi Arata, Tamahiro Kinjo

**Affiliations:** a Department of Cardiovascular Surgery, National Hospital Organization Kagoshima Medical Center, Kagoshima, Japan; b Department of Cardiovascular Surgery, Kagoshima City Hospital, Kagoshima, Japan

**Keywords:** Parkes Weber syndrome, Arteriovenous malformation, Embolization, Pseudoaneurysm, Amputation

## Abstract

A 63-year-old woman presented with a giant arteriovenous malformation (AVM) of the lower limbs associated with Parkes Weber syndrome (PWS). The AVM was supplied by 4 branches of the right profunda femoris artery and 4-stage embolization was performed. At 67 years of age, she developed a pseudoaneurysm due to the recanalization of a coiled profunda femoris artery branch arterial aneurysm. We performed re-embolization of the coiled profunda femoris artery and observed its shrinkage, but 2 months later, the pseudoaneurysm began to expand again, having a fatal course. This case indicates that battles against giant AVM-associated PWS follow a long and convoluted path. We present this case with reviewing 15 previously reported cases to improve our management of PWS.

## Introduction

Parkes Weber syndrome (PWS) is a rare genetic disease that manifests as red skin lesions, high-flow arteriovenous malformations (AVMs), and hypertrophy of the unilateral limb [1]. AVM is a progressive disease with manifestations ranging from the Schobinger classification of quiescence stage (I) to decompensation stage (IV) ([2], Table 1). Patients with AVM require invasive treatment after transitioning to the symptomatic stage. Embolization may be a safe and effective option for high-flow AVMs [3]. However, some unsuccessful cases have been reported and reports on the long-term follow-up after embolization are sparse. In this report, we present a patient with PWS whose staged embolization failed and resulted in an uncontrollable pseudoaneurysm. While true aneurysm has distended vascular wall, pseudoaneurysm has disrupted vascular wall and surrounding fibrous tissue wall. Moreover, we review 15 previously reported cases to improve our understanding of the management of PWS.

**Table 1:** Schobinger's classification.

Stage	Clinical features
Ⅰ (Quiescence period)	May or may not have vascular skin stain, warmth of the affected tissues, and arteriovenous shunts can be detected by Doppler ultrasound. The arteriovenous malformation is present but causes no clinical symptoms
Ⅱ (Expansion period)	Stage I plus enlargement, pulsations, palpable thrill, audible bruit, and enlarged arterialized tortuous/tense veins
Ⅲ (Destruction period)	Stage II plus dystrophic skin changes, skin ulcerations that can be nonhealing, bleeding from ulcerated skin or mucosal surfaces, overt tissue necrosis, and lytic lesions of bone may occur
Ⅳ (Decompensation period)	Stage III plus congestive cardiac failure with increased cardiac output, abnormally lowered peripheral vascular resistance, and venous hypertension secondary to tissue and skin changes

## Case presentation

**Fig. 1:**
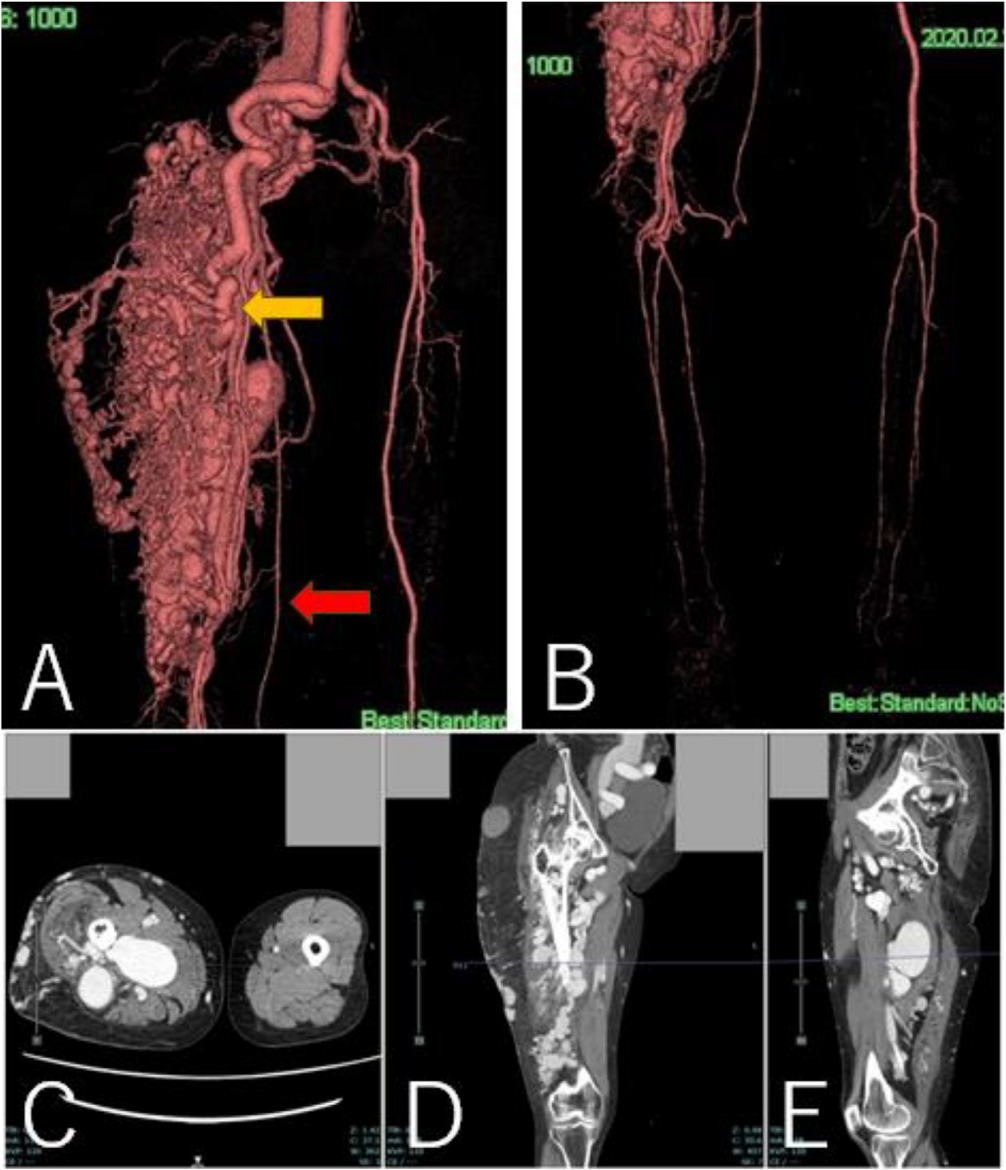
3D-CT angiography and multi-planar reconstruction of a giant arteriovenous malformation (AVM).(A) The feeding vessels included 4 branches from the right profunda femoris artery and the right superior and inferior gluteal arteries. One of the profunda femoris artery branches had a saccular arterial aneurysm (orange arrow, 20 × 17 × 27 mm). The right superficial femoral artery was hypoplastic and was not involved in the AVM (red arrow).(B) The giant AVM was located only in the right femoral region.(C) Axial view. (D) Coronal view. (E) Sagittal view.

**Fig. 2:**
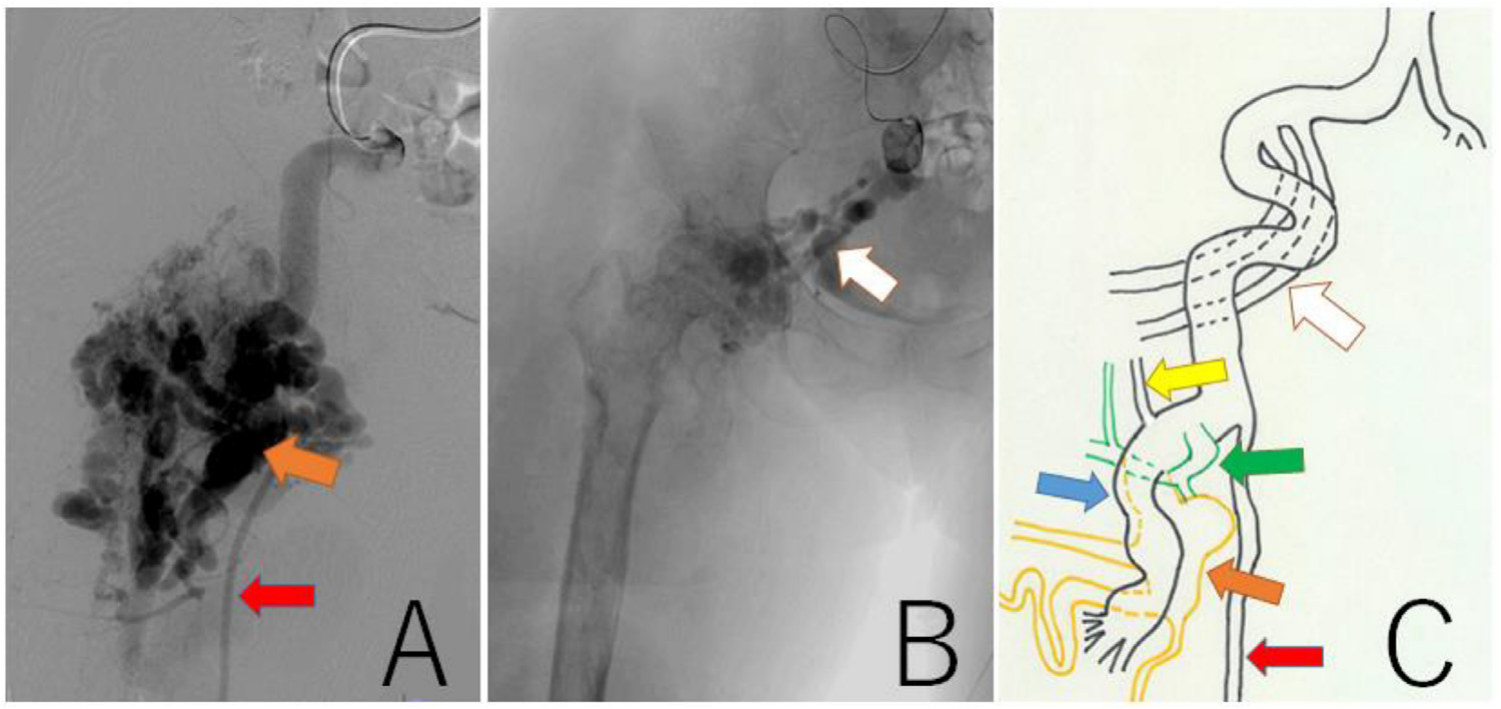
Findings of angiography and a scheme of AVM feeders. (A) Angiography from the external iliac artery showed that profunda femoris artery branches (B1-B4) were high flow feeders of the AVM. The B2 branch had a small saccular arterial aneurysm at its origin (orange arrow). The superficial femoral artery (red arrow) was hypoplastic. (B) Angiography from the inferior gluteal arteries (white arrow). (C) The AVM included 4 branches (B1: blue arrow, B2: orange arrow, B3: green arrow, B4: yellow arrow) of the profunda femoris artery, and superior and inferior gluteal arteries (white arrow).The red arrow is superficial femoral artery.

**Fig. 3:**
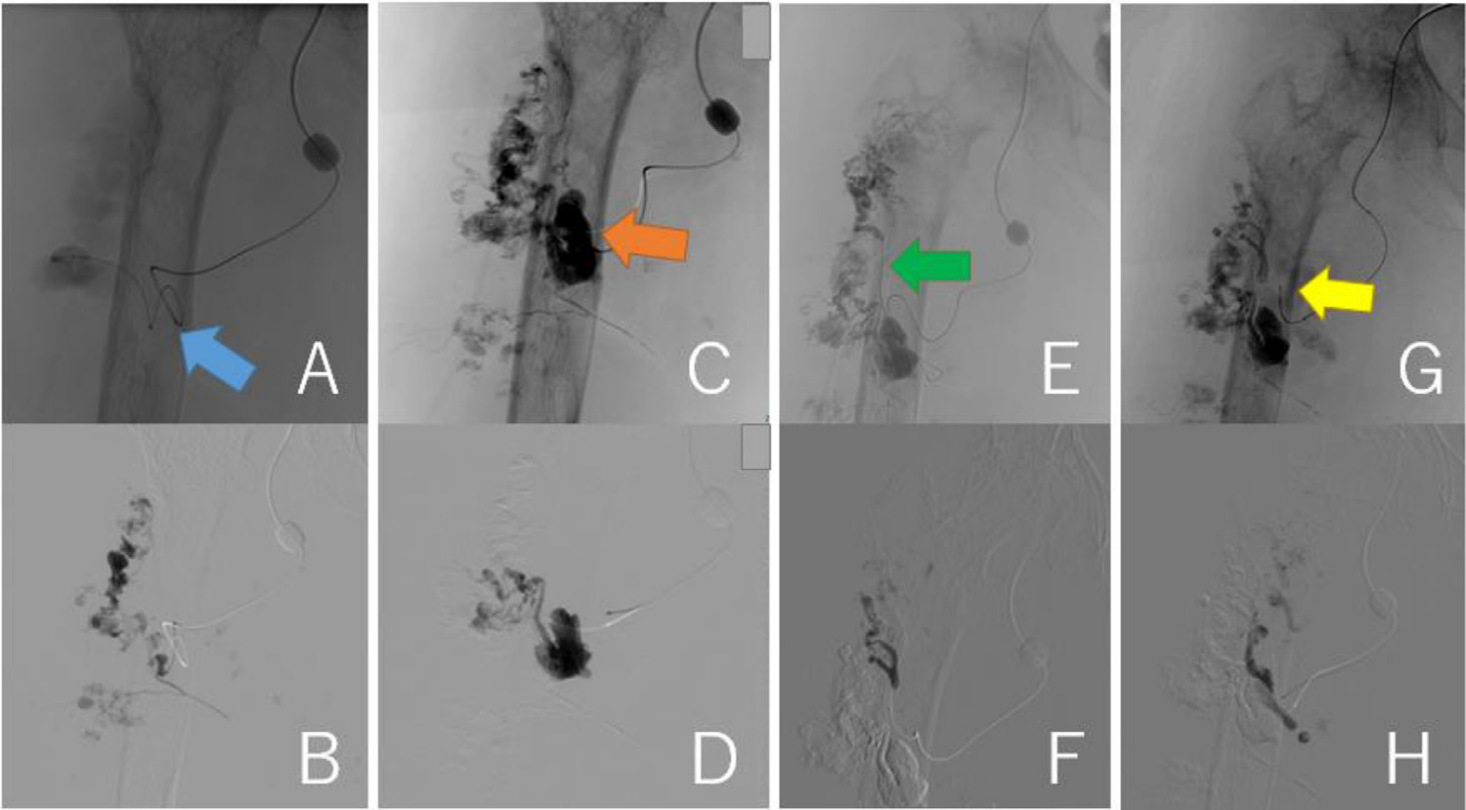
Findings of the first embolization with n-butyl-2- cyanoacrylate plus ethiodized oil. (A, B) B1 (blue arrow) was embolized. (C, D) B2 (orange arrow) was embolized. (E, F) B3 (green arrow) was embolized.(G, H) B4 (yellow arrow) was embolized.

**Fig. 4:**
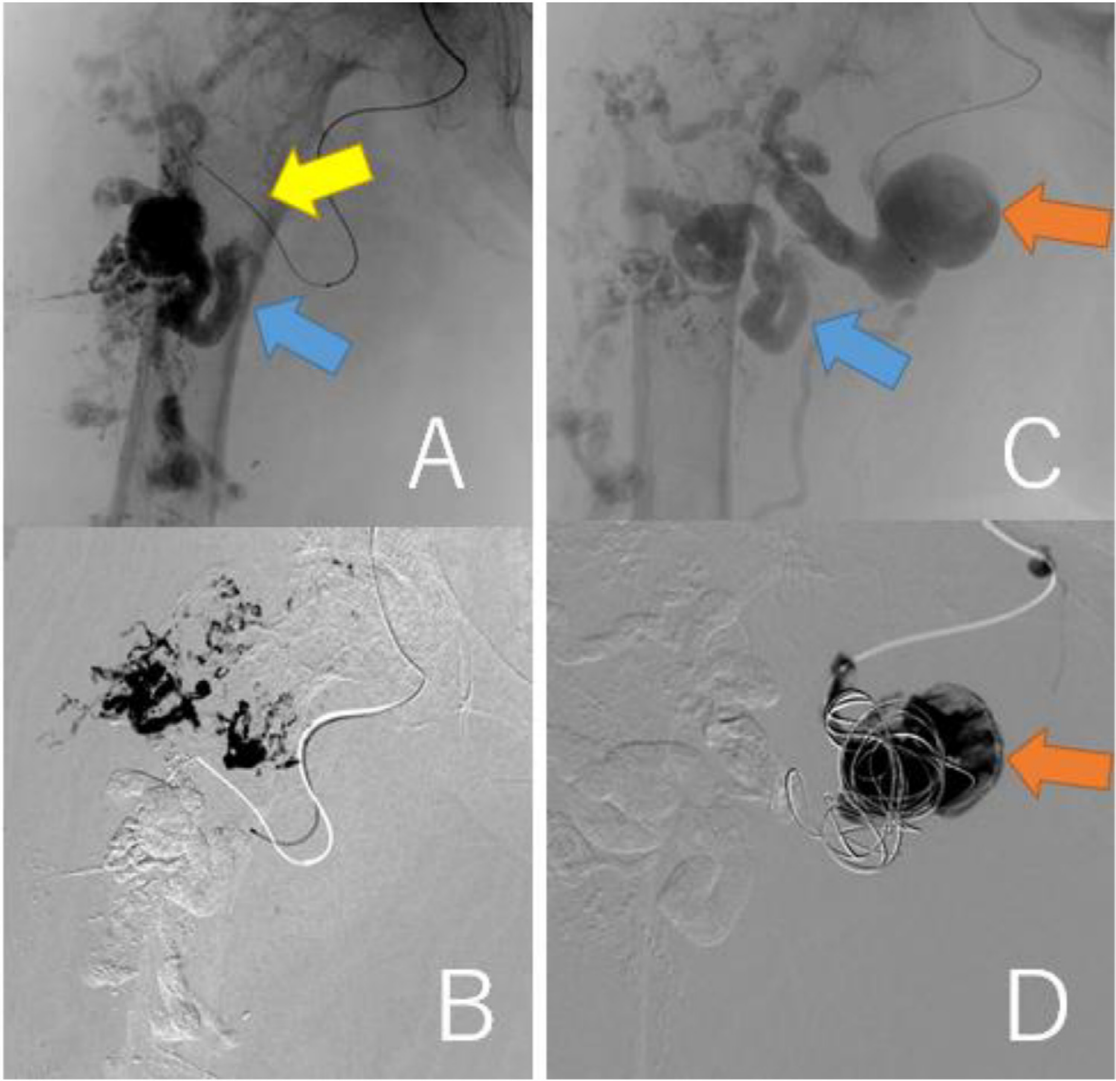
Findings of the third embolization. (A, B) B4 (yellow arrow) was embolized with NBCA plus ethiodized oil. Blue arrow represents B1. (C, D) A saccular B2 arterial aneurysm (20 × 17 × 27 mm, orange arrow) was embolized with Target® XXL360 coils (Stryker, Michigan, U.S.A.) (24 mm × 50 cm × 2) and NBCA plus ethiodized oil.

A 63-year-old woman (height, 147 cm; weight, 46.5 kg) with a history of subarachnoid hemorrhage (SAH) had pain in the right thigh and popliteal fossa during walking. She had hypertrophy in the right thigh since childhood and found a varicose vein a few years ago. The patient's family history was unremarkable. She consulted a physician, and contrast-enhanced CT revealed a giant AVM in the right thigh (Figs. 1A-E). The right common and external iliac artery was tortuous and dilated, and both superficial femoral arteries were hypoplastic. Ankle brachial index was 0.78 at the right leg and 1.09 at the left leg. She was then referred to a hospital specializing in AVM. The Schobinger classification was stage Ⅲ and she was diagnosed with PWS based on clinical features. Angiography showed that 4 branches (B1–B4) of the right profunda femoris artery, and superior and inferior gluteal arteries formed the AVM (Figs. 2A-C). The B2 branch had a small saccular arterial aneurysm at its origin. The AVM was a dilated fistula between multiple feeding arteries and draining veins, and was categorized as a type IIIb arteriovenous fistula according to Cho's classification [4]. Staged embolization was planned, and 3 months after the angiography, the first embolization was performed for the distal B1–B4 with n-butyl-2- cyanoacrylate (NBCA) plus ethiodized oil (Figs. 3A-H). Six months later, a second embolization was performed for B1 with NBCA and ethiodized oil. Furthermore, 6 months later, when she was 65 years old, the third embolization was performed for B4 with NBCA plus ethiodized oil (Figs. 4A and B) and for a saccular B2 arterial aneurysm (20×17×27 mm) with Target® XXL360 coils (Stryker, Michigan, U.S.A.) (24 mm×50 cm×2) and NBCA plus ethiodized oil (Figs. 4C and D). Complete occlusion of the aneurysm was confirmed. Seven months later, a fourth embolization was performed for a superior gluteal artery branch, using NBCA plus ethiodized oil. The other superior gluteal artery branches had low flow, and the inferior gluteal artery was difficult to embolize selectively. Embolization from the proximal profunda femoris artery was performed with NBCA plus ethiodized oil for finishing. Regular MRI follow-up 6 months later did not reveal any complications or more enlarged AVM.

**Fig. 5:**
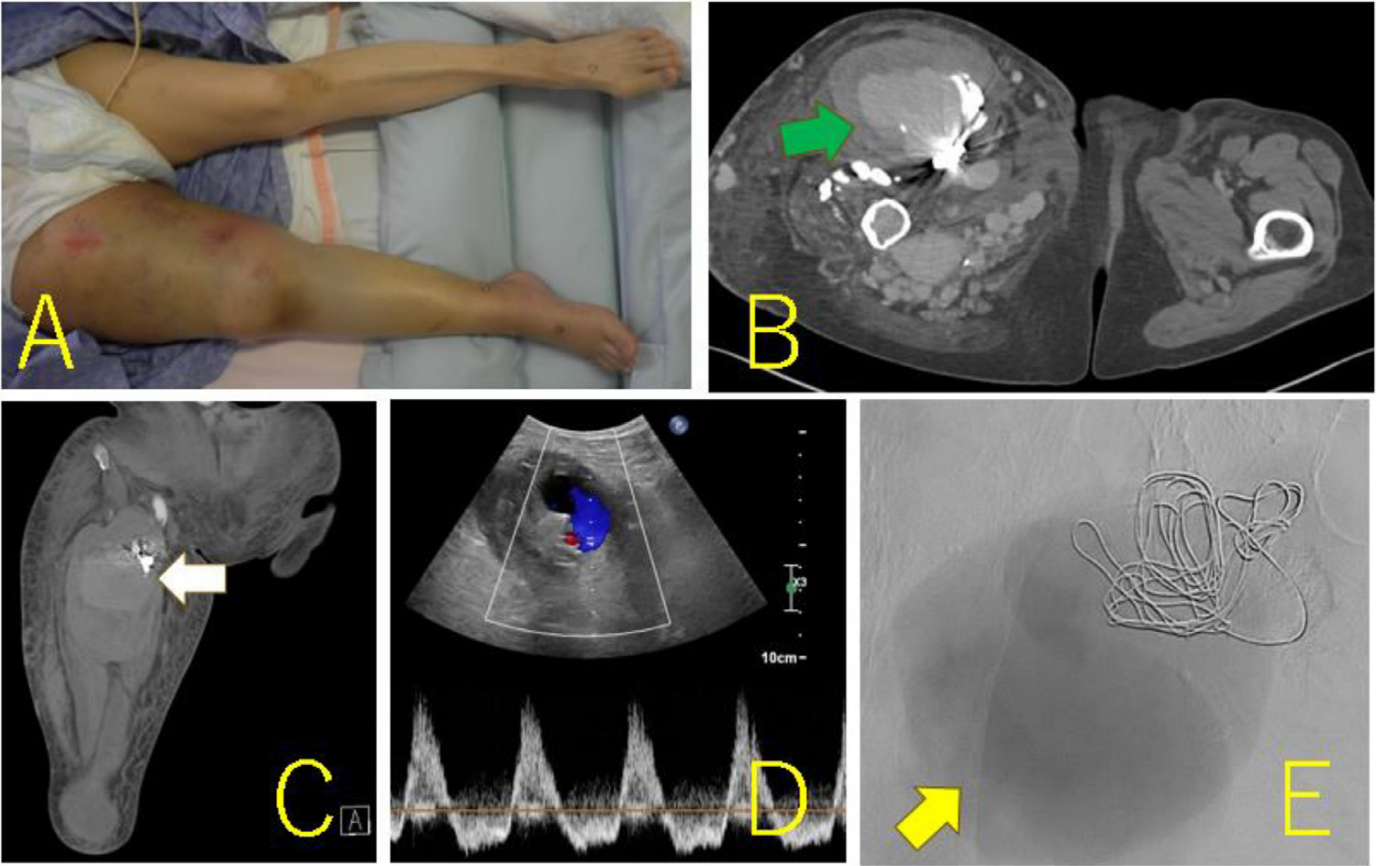
The photograph and imaging findings on admission to our hospital. (A) The right thigh was swollen, was twice as large as the left leg, and had red skin lesions. (B, C) Axial (B) and coronal (C) contrast-enhanced computed tomography revealing a giant aneurysm (green and white arrows) (97 × 71 × 154 mm) next to the coils. (D) Ultrasonography of the limb arteries shows a to-and-fro waveform between the giant aneurysm and the coiled B2 arterial aneurysm, suggesting that the giant aneurysm was a pseudoaneurysm. (E) Digital subtraction angiography image showing the pooling of the contrast agent in the giant pseudoaneurysm (yellow arrow).

At 66 years of age, she was transferred to our hospital with sudden swelling of her right thigh, edema of her right lower leg, and difficulty walking. Physical examination revealed that her right thigh was twice as large as her left thigh and had red skin lesions (Fig. 5A). The enlarged right thigh was also pulsating and hot. Her vital signs were within the normal limits.

Admission laboratory testing revealed a wide range of abnormalities, as follows: white blood cell and platelet counts, and hemoglobin level at 9330/μL, 71,000/μL, and 5.0 g/dL, respectively. The levels of the fibrin/fibrinogen degradation products were 106.1 μg/mL. The patient was diagnosed with disseminated intravascular coagulation (DIC). The blood urine nitrogen, serum creatinine, and C-reactive protein levels were 63.0 mg/dL, 3.30 mg/dL, and 14.32 mg/dL, respectively.

Contrast-enhanced computed tomography (CT) revealed a giant aneurysm (97×71×154 mm) adjacent to the embolization coils in the right thigh (Figs. 5B and C). Ultrasonography of the limb arteries revealed a to-and-fro blood waveform via a 5-mm channel between the giant aneurysm and the coiled B2 arterial aneurysm, suggesting that the giant aneurysm was a pseudoaneurysm (Fig. 5D). Chest X-ray revealed a cardiothoracic ratio of 0.60 and echocardiography revealed dilated ventricles.

The patient first received conservative therapy to improve her general condition, which consisted of bed rest, hydration, blood transfusion, compression of the enlarged right thigh, and elevation of the right lower limbs. Profunda femoris artery angiography revealed high flow, with contrast pooling in the giant aneurysm (Fig. 5E). Because the aneurysm had enlarged rapidly, we concluded that the coiled B2 arterial aneurysm had recanalized, ruptured, and generated a giant pseudoaneurysm in which red blood cells, platelets, and coagulation factors were consumed, leading to DIC.

**Fig. 6:**
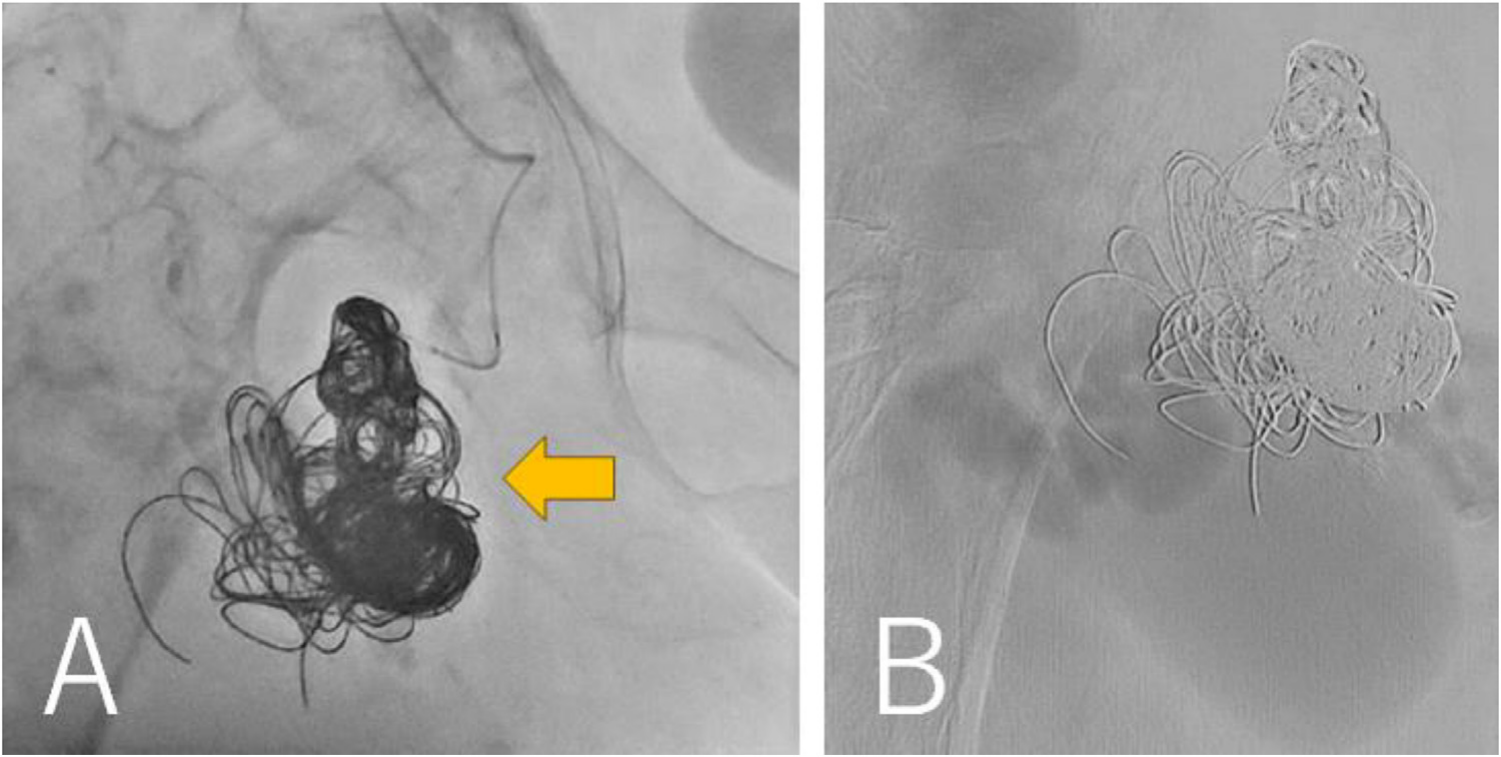
Findings during the latest embolization procedure. (A) The coiled B2 arterial aneurysm was embolized with additional 12 coils (orange arrow). (B) Digital subtraction angiography showed that the blood flow into the pseudoaneurysm disappeared.

**Fig. 7:**
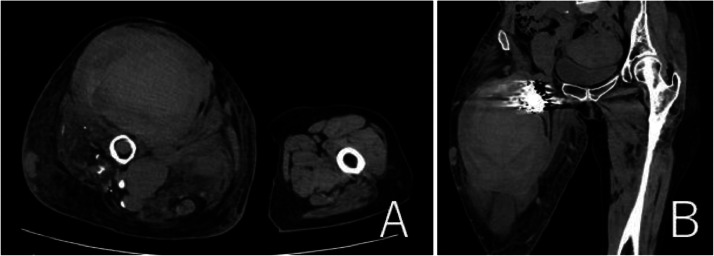
Findings of CT 2 month after discharge. (A, B) CT revealed more expanded pseudoaneurysm (131 × 107 × 180 mm).

She was conservatively managed in our hospital for 1 week, but the skin on her right thigh was tighter. CT showed further enlargement of the pseudoaneurysm (113 × 85 × 175 mm) and the diameter of the right thigh was 216 × 248 mm. We concluded that vascular surgical procedures would not control bleeding after incision of the pseudoaneurysm. The patient subsequently underwent re-embolization of the coiled B2 arterial aneurysm via the left femoral artery. The right profunda femoris artery flow was rapid, necessitating the use of 11 Target ® XXL360 coils(24 mm × 50 cm × 4, 22 mm × 50 cm × 4, 20 mm × 50 cm × 1, 16 mm × 50 cm × 1, 10 mm × 50 cm × 1)and 1 Interlock® (Boston Scientific, Massachusetts, U.S.A.) coil 14 mm × 50 cm (Fig. 6A). Digital subtraction angiography revealed that the contrast agent was not visible in the giant pseudoaneurysm after the procedure (Fig. 6B). The pulsation in the right thigh disappeared. One week after the procedure, platelet counts and hemoglobin levels recovered to 261,000/μL and 9.1g/dL, respectively. Contrast-enhanced CT revealed shrinkage of the giant pseudoaneurysm (119 × 65 × 173 mm) and the diameter of the right thigh (201 × 225 mm). Ultrasonography also revealed the absence of blood flow into the re-embolized B2 arterial aneurysm and a giant pseudoaneurysm. The patient was discharged to her referring hospital 14 days after the procedure. However, 2 months after the transference, the right thigh began to swell and involved skin ulceration. CT revealed an expanded pseudoaneurysm (131 × 107 × 180 mm, Figs. 7A and B). She was given the option of an additional procedure but chose conservative therapy. The ulcerated right thigh was applied with KALTOSTAT^Ⓡ^ Alginate Dressing (Convatec, New Jersey, USA) and was compressed. The ulcerated wound involved oozing and severe bacterial infection, and 7 months after the transference she died.

## Discussion

PWS often occurs sporadically and is associated with loss-of-function mutations in the *RASA1* gene [5], which encodes the p120-Ras GAP protein responsible for controlling cellular proliferation and differentiation [6]. The *RASA1* gene is also associated with cerebral AVMs [4], and spinal AVMs are associated with PWS [7]. The patient's past SAH was not caused by a cerebral AVM but by a posterior communicating internal carotid artery aneurysm. A skin biopsy of the affected limb could have revealed a pathogenic variant of *RASA1* [8]; however, this was not performed in this patient.

In Japan, giant AVM in a limb has been designated by the Ministry of Health, Labour, and Welfare as an “intractable disease.” The estimated number of Japanese patients with giant AVM in a limb is 700 [9] and the Japanese prevalence rate is 0.56 patients per 100,000 people. Therefore, we searched PubMed for case reports of giant AVM associated with PWS to improve our understanding. We found 15 case reports and 15 clinical cases stage Ⅲ and Ⅳ of Shobinger's classification when we searched with the terms ‘‘(Parkes Weber Syndrome)’’ (Table 2). The number of female patients was almost equal to the number of male patients (7 female cases and 8 male cases). In 2 cases [19,23] stage Ⅳ AVM was observed in the neonate. The most common AVM site was the lower limb (11 lower limb cases and 4 upper limb cases), and no bilateral lesions were reported. Embolization [12,13,16,[19], [20], [21],^24^] was the most common therapy for giant AVM, but it was not effective in more than half cases [13,16,21,24].

**Table 2:** Summary of 15 reported cases of a giant AVM associated with Parkes Weber syndrome.

Author, country	Age/Sex	AVM Site	Previous AVM treatments	Symptoms and conditions	Treatments	Outcomes
Yamamoto, Japan [10]	23/M	Right lower leg	-	Lower leg fracture	Cast immobilization	Union of the fractures at 2 years and 3 months after injury.
Dubrey, U.K. [11]	21/F	Right thign and leg	Surgical interventions	Skin bleeding	Compressive therapy	Unknown
Plasencia, U.S.A. [12]	37/M	Right upper arm	-	Skin ulcer	Embolization	Uneventful, but long term follow-up is unknown
Yahata, Japan [13]	23/M	Left leg	Embolization	Limb ischemia and skin ulcer	Amputation	Uneventful more than 2.5 years
Stefanov-Kiuri, Spain [14]	77/F	Left forearm	-	Skin ulcer	Compressive therapy and local wound care	Unknown
Kondapavuluri, India [15]	32/F	Right leg	-	Skin bleeding ulcer	Disarticulation	Unknown
Espitia, France [16]	61/M	Left thigh	-	Heart failure	Embolization	Death at day 40
Chagas, Brazil [17]	21/F	Left leg	Surgical intervention	Skin ulcer, joint pain and claudication	Compressive therapy	Uneventful, but long term follow-up is unknown
Sharma, India [18]	17/M	Left thigh to leg	-	Skin plaques, varicose veins and erosions	Compressive therapy and topical emollients	Improvement after 1 month of follow-up
Anzina, Latvia [19]	Neonate /F	Left scapula and shoulder	-	Heart failure	Embolization of AVM and occ|usion of PDA	Stable at the age of 16 months.
Zhao, China [20]	18/F	Right leg	-	Hypertrophic stains	Embolization	No sign of recurrence 2 years after the procedure
Acar, Turkey [21]	15/M	Left upper arm	Embolization	Heart failure	Implantation of covered stents	Recurrence of heart failure 6 months after the intervention
Patel, U.S.A. [22]	20/F	Left thigh	-	Pain of the thigh	Analgesics	Unknown
Peñalver, U.S.A. [23]	Neonate/ M	Left upper arm	-	Heart failure	Surgical banding	Improvement over the course of several months.
Yamamoto, Japan [24]	51/M	Right buttock and pelvis	Surgical ligation and embolization	Heart failure	Embolization	Death 1.5 years later
Present case, Japan	67/F	Right thigh	Embolization	Sudden swelling of the thigh and walking difficulty	Embolization of the aneurysm next to a pseudoaneurysm	Re-expansinon of the pseudoaneurysm 2 months after embolization

AVM, arteriovenous malformation; PDA, Patent ductus arteriosus.

PWS is a progressive disease with a poor prognosis. Therefore, multidisciplinary therapy is needed for the management of giant AVM. Patients in the quiescent stage (I) or expansion stage (II) of the Schobinger clinical classification have fewer symptoms and are often treated conservatively with bed rest, elevation, and compression of the affected limb. In contrast, patients in the destructive stage (III) and decompensation stage (IV) have severe and life-threatening conditions, and invasive treatments such as sclerotherapy, embolization of the feeding arteries and draining veins [3], and major amputation or disarticulation of the affected limb [15] are required. During embolization, the feeding arteries should be occluded distally close to the shunt and not proximal to the shunt. Otherwise, the shunt may remain patent, and collateral recruitment may develop into a shunt. Thus, embolization should be as selective as possible for shunts where the feeding arteries and drainage veins are abnormally connected. Amputation should be considered in cases of unsuccessful embolization. Amputation of the limb affected by a giant AVM can cause massive hemorrhage. Ismail [25] and Young [26] employed partial cardiopulmonary bypass to minimize hemorrhage.

Reports of pseudoaneurysm formation after coil embolization and persistent pseudoaneurysm are few. Pathological dilated vascular wall [24] of AVM or radial force of coils might induce recanalization of coiled aneurysm. The unleashed edge of coil might disrupt the fragile vascular wall, leading to rapid growth of pseudoaneurysm. Moreover, collateral vessels or migration of the embolized coils might help develop persistent pseudoaneurysm [27]. Pseudoaneurysm can be diagnosed by ultrasound. A swirling pattern inside the false lumen by the color-flow image, and ‘‘to-and-fro’’ waveform at the communicating point between the native vessel and the false lumen are characteristic of pseudoaneurysm [28]. Contrast enhanced CT can distinguish pseudoaneurysm from ruptured aneurysm. While the contrast increases and changes its shape in the active bleeding, pseudoaneurysm maintains its shape on delayed phase. Moreover, pseudoaneurysm may not always fill with contrast due to the presence of thrombus [29].

Our patient's giant AVM was fed by the profunda femoris artery and superior and inferior gluteal arteries, and invaded the right ischial bone. If this patient had wished for an additional procedure, re-embolization would not have controlled the life-threatening giant pseudoaneurysm again, so we would have proceeded to perform an orthopedic procedure as a last resort. In this case transfemoral amputation and hip disarticulation are challenging because the pseudoaneurysm is located proximal to the pelvis and the procedure involves complex vascular ligation techniques with massive hemorrhage. Therefore, we would have performed transpelvic hemi-amputation (hemipelvectomy). This procedure is invasive and technically demanding, but it can ligate the unilateral common iliac artery and vein safer [30] and eradicate both the pseudoaneurysm and giant AVM.

Herein, we describe a rare case of pseudoaneurysm formation after embolization of a giant AVM of the lower limb associated with PWS. Embolization may be a safer and more effective option than surgery for high-flow AVMs [3] in terms of pain reduction and ulcer healing. However, as shown in Table 2, the effectiveness of embolization may be temporary. Repeated interventions are generally required for PWS, and strategies against PWS follow long and convoluted paths. The pathological findings of AVMs reveal that the malformed arterial wall consists of heterogeneous elastic fibers [31], indicating the possibility that AVMs and aneurysms can enlarge over time, even after embolization. When embolization of a giant AVM is ineffective, amputation and disarticulation of the affected limb should be considered as an eradication therapy.

## Conclusion

The treatment of a giant AVM of the lower limb associated with Parks follows a long and convoluted path. When embolization is ineffective, an orthopedic procedure should be considered.

## Author contributions

Study conception: HY

Data collection: HY

Writing: HY, KT, TK

Critical review and revision: All authors.

Final approval of the article: All authors.

Accountability for all aspects of the work: All authors.

## Patient consent

The patient's identity was protected, and informed consent was obtained from the patient for the publication of this report.
